# Editorial: Challenges in Acute Minor Ischemic Stroke

**DOI:** 10.3389/fneur.2022.896716

**Published:** 2022-04-06

**Authors:** Mirjam R. Heldner, Pierre Seners, Linxin Li

**Affiliations:** ^1^Department of Neurology, University Hospital Bern, Bern, Switzerland; ^2^Fondation Ophtalmologique Adolphe de Rothschild, Paris, France; ^3^Wolfson Centre for Prevention of Stroke and Dementia, Oxford University, Oxford, United Kingdom

**Keywords:** minor stroke, treatment, prognosis, epidemiology, thrombolysis, endovascular treatment

More than half of acute ischemic stroke patients can present with minor symptoms, yet they don't necessarily all have a benign outcome with up to a third of these patients having a modified Rankin Scale of 2 or more at 90 days. In this series, we have focused on various challenges in managing patients with acute minor ischemic stroke, ranging from acute treatment to prognosis.

Whilst all our contributors should be congratulated on their excellent and in some cases very delicate work in the area, it is worth noting that various operational definitions of minor stroke have been and are still being used. The two most commonly used cut-offs were NIHSS ≤ 5 in hyper-acute settings and NIHSS ≤ 3 in TIA/minor stroke or in secondary prevention settings more generally.

One of the biggest challenges in managing patients with acute minor ischemic stroke is perhaps the decision for acute reperfusion therapies such as intravenous thrombolysis (IVT) or endovascular treatment (EVT). With regards to IVT, as summarized by the two comprehensive reviews from Ferrari et al., and from Slawski and Heit, decisions to treat are likely to be made weighing the potential gain of improved functional outcome against the increased risk of intracerebral hemorrhage on an individual basis. An overall benefit is more likely in those presenting with disabling symptoms or those with proven large vessel occlusion (LVO). Whilst an increase of IVT in minor ischemic stroke has been observed in high-income countries in recent years, we still have a number of areas to improve, including better pre-hospital recognition of such patients, ultimate choice of thrombolytic agent (Alteplase vs. Tenecteplase) and identifying patients with early neurological deterioration. Indeed, Tang et al. showed in their single center experience that about 10% of the patients who received IVT after presenting with a minor stroke still experienced early neurological deterioration, which were associated with high baseline systolic blood pressure and history of coronary heart disease.

Another well-recognized factor that is associated with early neurological deterioration and poor outcome in minor ischemic stroke is the presence of LVO, which leads to the discussion about the role of EVT, another aspect of acute reperfusion therapies. LVO is not rare as demonstrated by Duloquin et al. Using data from the well-established Dijon Stroke Registry, the authors found that ~4% of patients with a mild ischemic stroke had LVO. Interestingly there was little evidence of any obvious predictors for LVO, showing the importance of timely arterial imaging in patients who presented with mild symptoms. With regards to EVT in such patients, Volbers et al. found in a retrospective cohort study that patients with minor deficits and LVO tended to have worse outcome compared to patients who presented initially with more severe deficits, especially in the context of secondary neurological deterioration, which was strongly associated with more proximal occlusion, suggesting that preventing such deterioration with EVT in high-risk patients might be one important way to improve functional outcome. They also showed that the approach starting with medical management first with rescue EVT after secondary neurological deterioration was associated with a poorer outcome, highlighting the importance of ongoing trials looking at immediate EVT vs. best medical treatment in patients with minor ischemic stroke and LVO, such as the Endovascular Therapy for Low NIHSS Ischemic Strokes (ENDOLOW) trial and the Minor Stroke Therapy Evaluation (MOSTE) trial. Whilst we are still waiting for the trial results, it was encouraging to see some positive trends from prospective cohort studies, such as the study led by Liu et al. They found that patients with mild symptoms and acute LVO in the anterior circulation had a higher proportion of independent outcome if they received EVT compared to those that were treated medically.

Of course, a large proportion of patients with minor ischemic stroke do not necessarily require acute reperfusion therapy and for those with non-cardioembolic events, short-term use of dual antiplatelet treatment, commonly Aspirin and Clopidogrel, is recommended by guidelines. However, 10–20% of patients still had early recurrence despite being on dual antiplatelet therapy, partly due to high residual on-treatment platelet reactivity (HRPR). Whilst there have been very promising data on genetic testing to identify CYP2C19 loss-of-function carriers, who are likely to be Clopidogrel non-responders, clinical markers remain extremely helpful. Guo et al. showed elegantly that in acute ischemic stroke patients taking dual antiplatelet treatment, history of diabetes might be such a marker. They found that diabetes was associated with increased platelet reactivity and higher prevalence of HRPR to Clopidogrel. If proven in other larger and prospective studies, their research offers a potentially simple approach for more personalized treatment in the future.

Ultimately the most important aspect in managing minor stroke is to improve the short and longer term prognosis. On one hand, it was disappointing to see that the onset to door time was prolonged during the COVID-19 pandemic in patients presenting with transient ischemic attack (TIA) or minor stroke in some Japanese hospitals, as shown by Tanaka et al. Their research reminds us that better public education on recognition of more minor events is still urgently needed. On the other hand, it was encouraging to see from the Australian community-based study, The INternational comparison of Systems of care and patient outcomes In minor Stroke and TIA (INSIST) study led by Tomari et al., that perhaps owning to early implementation of antithrombotic treatment, the 1-year risk of stroke in patients with TIA or minor stroke in their region was lower than previously reported.

Whilst we have made great progress in improving outcomes after minor strokes, there is certainly still room for further improvement and future potential new treatment targets are always welcomed. Li et al. measured heart rate variability on ECG, which is a marker for autonomic function, and showed that low heart rate variability was associated with higher stroke recurrence and worse functional outcome at 90 days after TIA or minor stroke. More research is still needed to determine if autonomic function can be a potential new treatment target. Tan et al. investigated efficacy and safety of adherence to dl-3-n-Butylphthalide (NBP) treatment in patients with non-disabling minor stroke and TIA and found that compliance with NBP therapy was associated with better 90-day functional outcomes particularly in patients presenting with minor stroke, although there was some unexplained signal of an increased risk of recurrent stroke in the NBP compliant group, which warrants further research.

Finally, in additional to more conventional approaches mentioned above, health innovations and technology is also evolving in the area. A good example is illustrated by Wijesundera et al., who showed that vision and visuomotor performance can be rapidly measured with bedside iPad apps after minor stroke. Hopefully with continued efforts as demonstrated in this series of research, we will be able to do better at managing patients with minor stroke in the very near future.

## Author Contributions

LL wrote the initial draft and all authors contributed to the conceptualization and revision of the manuscript. All authors contributed to the article and approved the submitted version.

## Conflict of Interest

The authors declare that the research was conducted in the absence of any commercial or financial relationships that could be construed as a potential conflict of interest.

## Publisher's Note

All claims expressed in this article are solely those of the authors and do not necessarily represent those of their affiliated organizations, or those of the publisher, the editors and the reviewers. Any product that may be evaluated in this article, or claim that may be made by its manufacturer, is not guaranteed or endorsed by the publisher.

